# Specificity and flexibility in sensory substitution: learning to grasp with vibration on one or two fingers

**DOI:** 10.3389/fpsyg.2026.1674806

**Published:** 2026-09-03

**Authors:** Saroosh Bilal, Raoul M. Bongers, David M. Jacobs

**Affiliations:** 1 Facultad de Psicología, Universidad Autónoma de Madrid, Madrid, Spain; 2 University Medical Center Groningen, University of Groningen, Groningen, Netherlands

**Keywords:** active perception, functional organization, information, perceptual system, reaching and grasping, sensory substitution glove, transfer of learning

## Abstract

Sensory substitution gloves (SSGs) are gloves that provide stimulation to the hand based on information such as the distance to objects in the environment. In recent years, it has been shown that SSGs enable users to interact with objects in the environment, including the grasping of objects. Important for the current study, different configurations of SSGs have been tested, and performance with SSGs has been shown to improve with practice. This led to the main question of our study: Does practice with one configuration of an SSG transfer to another configuration? The SSG that we used had actuators on the index finger and thumb that could vibrate when a finger pointed in the direction of a target. The vibration was more intense if the finger was closer to the object. Two modes of functioning were used: with vibration only on the index finger or with vibration on both fingers. In the reported experiment, 58 participants were divided into two groups that practiced a reaching and grasping task using the SSG either in the one-finger or two-finger mode. Before and after practice, both groups were tested in both conditions. Although average performance was better with two fingers than with one, substantial improvements were observed in both conditions, indicating a large amount of bidirectional transfer. These results are consistent with the interpretation that training with one or two fingers are different ways to improve the same functional system.

## Introduction

Sensory substitution devices (SSDs) are devices that allow individuals to perceive or act based on artificially produced stimulation presented through sensory modalities other than the ones that are typically used for those perceptions and actions. SSDs are often aimed at individuals with a loss or impairment of a sense modality (Elli et al., 2014). In the case of a visual impairment, for example, visual information can be transmitted through touch (Cancar et al., 2013; Froese et al., 2012; Lobo et al., 2018) or hearing (Abboud et al., 2014; Meijer, 1992). A famous SSD, known as the Tactile-Visual Sensory Substitution device (TVSS), was introduced by Bach-y-Rita et al. (1969). The original TVSS captured visual information with a camera and presented tactile stimulation to the user’s back. Since the TVSS, numerous visual-to-tactile SSDs have been developed (Eagleman and Perrotta, 2023; Ptito et al., 2021).

Among the currently available SSDs is the subgroup of sensory substitution gloves (SSGs). A precursor of the SSGs can be found in Lenay et al. (1997). These authors attached a light-sensitive sensor to a finger of the right hand. If the finger pointed in the direction of an object (in this case a light source), vibration was applied with a single vibrotactile actuator. In line with the work by Lenay et al. (1997), de Paz et al. (2023) developed a glove with two vibrotactile actuators, placed on the dorsal sides of the index finger and thumb. These actuators vibrated when the respective fingers pointed in the direction of a target object, with more intense vibration for nearer objects. As indicated by Lenay et al. (1997), information about the position of objects can be obtained from the pointing-vibration relations that emerge when individuals use the devices. Other recent SSGs include the ones by Kilian et al. (2022; with a 3 × 3 array of vibrotactile actuators), Teng et al. (2025; with a 5 × 6 array of electrotactile actuators), and Tajdari et al. (2025; with 4 vibrotactile actuators).

In this quickly growing body of research, therefore, different configurations of SSGs with one, two, or more actuators have been proposed, developed, and tested, using different grasping, reaching, and pointing tasks. Reasonably high levels of performance have been observed in the majority of the studies. It has also been shown that performance with SSGs improves after practice (Bilal et al., 2025a; Kilian et al., 2022). The fact that SSGs with different configurations have been proposed raises the question of the extent to which practice with one configuration of an SSG transfers to other configurations. This question is the focus of the current article. The question can be connected in interesting ways to studies about the transfer of learning and calibration that have been performed in the context of the ecological approach to perception and action (Gibson, 1966, 1979). To explain this connection, we need a brief review of some key concepts of the ecological approach.

In his seminal work, Gibson (1966) coined the term *perceptual systems*, referring to intentional systems that actively detect (or *hunt for*) information rather than respond in more passive ways to imposed stimulation. The goal of perceptual systems is to detect invariant information that specifies properties of the environment that are meaningful to the observer (Gibson, 1979). In this view, information may be multimodal and of a higher order, meaning that it may include, for example, visual, tactile, and proprioceptive components, as well as abstract relations within and among these (Stoffregen and Bardy, 2001). If detected and used, the information also comes to specify aspects of the performed actions (Michaels and Carello, 1981). In summary, environmental properties are specific to higher-order information, which, in turn, becomes specific to aspects of perception and action. One may therefore say that perceptual systems are *functionally specific*.

The functional specificity of perceptual systems is complemented by flexibility. For example, when certain components are constrained, perceptual systems can adaptively be reconfigured to detect the same information. Bongers and Michaels (2008) examined this adaptivity for judging and catching fly balls. Useful information in this situation is provided by the optical acceleration of the ball (Chapman, 1968). To detect optical acceleration, balls are usually tracked with eye and head movements (Oudejans et al., 1999). In their experiment, Bongers and Michaels restricted either one of these movements, or both. They observed that, as long as one type of movement was unrestricted, participants achieved high accuracy. This demonstrates that perceptual systems can detect information in a flexible manner: If one component is constrained, other components are recruited to achieve the same goal.

Another example of the flexibility of perceptual systems can be found in the literature on dynamic touch. In this paradigm, individuals perceive properties of (unseen) wielded objects, such as the length of hand-held rods. The information that is exploited in this task is related to the moments of inertia of the wielded objects, and, thereby, to detectable patterns in the muscles and tendons used during the wielding (Turvey, 1996; Solomon and Turvey, 1988). Importantly, object properties can be perceived through wielding with the dominant hand, non-dominant hand, or both hands (Carello et al., 1992); foot (Hajnal et al., 2007); torso (Palatinus et al., 2011); or head (Wagman et al., 2017). This indicates that the same moments of inertia can be detected from perceptual flows involving different anatomical units, hence demonstrating functional specificity as well as anatomical flexibility (cf. Duffrin and Wagman, 2024).

One might then ask: If one learns to perceive a property through dynamic touch, does one learn to perceive the property in a general or a particular way? More specifically, does one learn to detect the moments of inertia with any limb or only with the limb used in practice? If the former is the case, transfer of learning should be expected from one limb to others. If the latter is the case, no such transfer should be expected. Experimental data support the former. The currently most accepted explanation for this finding is that, during calibration and learning in dynamic touch, the functional system is adapted as a whole, not a particular way to detect the inertia-related information (Stephen and Hajnal, 2011; Withagen and Michaels, 2004).

We can now formulate the main question of this article. As indicated above, configurations of SSGs have been tested with vibration on one finger or two fingers. In the current study, we investigate whether learning to reach for and grasp objects with vibration on one finger transfers to performance with vibration on two fingers, and vice versa. In analogy to the research on dynamic touch and fly ball catching, this question has interesting implications. If no transfer is observed, what people learn are configuration-dependent strategies. If bidirectional transfer is observed, in contrast, the learning is in relevant ways independent of the configuration of the SSG. Learning could be configuration independent, for example, if learners come to exploit information about the object position from pointing-vibration relations that are largely a consequence of geometrical laws, and hence apply to all fingers. More technical hypotheses about such informational patterns are considered in the Discussion. Note, for the moment, that such patterns require a combination of pointing and vibration; the object position is not specified by the pointing alone and neither by the vibration alone.

In the experiment, a pretest-practice-posttest design was used. Participants were divided into two groups: the *one-finger group* practiced reaching for and picking up an object with vibrotactile stimulation on one finger and the *two-finger group* with vibrotactile stimulation on two fingers. Both groups performed the pretest and posttest with vibrotactile stimulation on one and on two fingers. With regard to our main question, we expected a substantial amount of transfer between the practice conditions, which may indicate that a flexible exploitation of abstract pointing-vibration relations is a key characteristic of SSG-based performance. This expectation is based on the flexibility in information detection that has been demonstrated in other tasks (Bongers and Michaels, 2008; Withagen and Michaels, 2004). Moreover, based on previous research, we expected that the grasping performance would already be reasonable in the pretest (Lenay et al., 1997; de Paz et al., 2023) and that it would further improve with practice (Bilal et al., 2025a). In addition to the performance and the transfer thereof, we analyzed measures related to exploration and the effectiveness of exploration.

Apart from the more theoretical implications, the experiment is of relevance from an applied perspective. Potential SSD users frequently report a preference for technological devices that offer minimal yet sufficient information and that are lightweight, robust, and affordable (Bilal et al., 2025b; Elli et al., 2014). In this sense, an SSG with vibration on one finger—if sufficiently effective—may have advantages over an SSG with vibration on two fingers.

## Method

### Participants

Fifty-eight right-handed participants (*M*_age_ = 19.8 years; *SD* = 4.7) were recruited and randomly assigned to the one-finger or two-finger group (*n* = 29 for each group). Of the participants, 82.8% were females and 17.2% were males. All of them were students at the Faculty of Psychology of the Autonomous University of Madrid. They received course credits for their participation. All participants had normal or corrected-to-normal vision and they did not report movement problems. The local ethics committee approved the experiment (UAM-CEI-125-2563). All participants provided written informed consent. The number of participants was determined with an *a priori* power analysis that was conducted with GLIMMPSE (Kreidler et al., 2013). The analysis focused on the three-way interaction in a mixed factorial design and was based on the effect size and variability estimates of Bilal et al. (2025a). Using a conditional power method and assuming a Type I error rate of 0.05, the analysis indicated that 58 participants would result in a power of 80.1%.

### Apparatus

The used SSG consisted of a thin and stretchable right-hand glove. Two vibrotactile motors were attached to the glove, one on the back of the index finger and one on the back of the thumb. The motors were coin-style eccentric rotating mass motors (VPM-2) with a diameter of 12 mm and a height of 3.4 mm, purchased from Active Robots (Radstock, United Kingdom). The motors were wired to a printed circuit board, which was further connected to a digital/analogue conversion card and a PC (Intel Core i7, 3.07 GHz). MATLAB (MathWorks, Inc., Version R2022b) was used to control the computer output and hence the vibration intensity of the motors.

Six infrared-reflective markers were attached to the glove. These markers were located on the distal interphalangeal, proximal interphalangeal, and metacarpophalangeal joints of the index finger and thumb. A seven-camera movement-capture system (Qualisys AB) tracked the locations of these markers at 100 Hz. To compute the vibration intensity of a motor, the MATLAB programs used the top two markers on the finger on which the motor was placed. These markers allowed the programs to trace an imaginary line in the pointing direction of the finger. If the imaginary line did not intersect the target, or if the distance between the tip of the finger and the target exceeded 45 cm, no vibration was applied. The motor did vibrate whenever the imaginary line intersected the target (i.e., whenever the finger pointed to the target) and the distance was smaller than 45 cm. The vibration intensity depended on the distance, ranging from its minimum at 45 cm to its maximum at 0 cm. This implies that when the fingertip was closer to the object, the vibration was stronger (and vice versa). If the distance *d* was between 0 and 45 cm, the formula used to set the signal that drove the vibration *V* (as a percentage of its maximum) was: $V=40+60\;{\left(\frac{45-d}{45}\right)}^{3}$ , leading to just noticeable vibration when *d* was 45 and intense vibration when *d* approached 0.

The quantity *V*, used in the previous paragraph, is proportional to the voltage sent to the motors. In addition to the voltage, the frequency and amplitude of vibration of a motor as a whole depends on how the motors are included in an SSD (e.g., how tight an SSG presses the motor to the finger in a particular position). Previous work with the same type of motors included in a different SSD showed that when *V* increased from 40 to 100, the frequency of the motors increased from 50 to 64 Hz (Figure A1 of Díaz et al., 2012). Díaz et al. also stated that the amplitude of the motors increased with *V*. Furthermore, they showed that individuals can detect increases of about 10–15 *V* if one applies the range (and scale of *V*) of our experiment (see their Figure A2). Given that Díaz et al. applied vibration to the torso and we to the fingertips, the sensitivity to increases in *V* of our participants should be assumed to be at least as good.

To allow the online computations of the distance that is needed to set *V*, the Qualisys software identified the markers before their locations were sent to MATLAB. This was done with an automatic identification of markers (AIM) model. The AIM model was created with the data of three individuals (not participants in the actual experiment) that performed several reaching and grasping movements similar to the ones in the actual experiment. The labelling of the markers with the AIM model was applicable also to the trajectories of the participants in the actual experiment.

**Figure 1:**
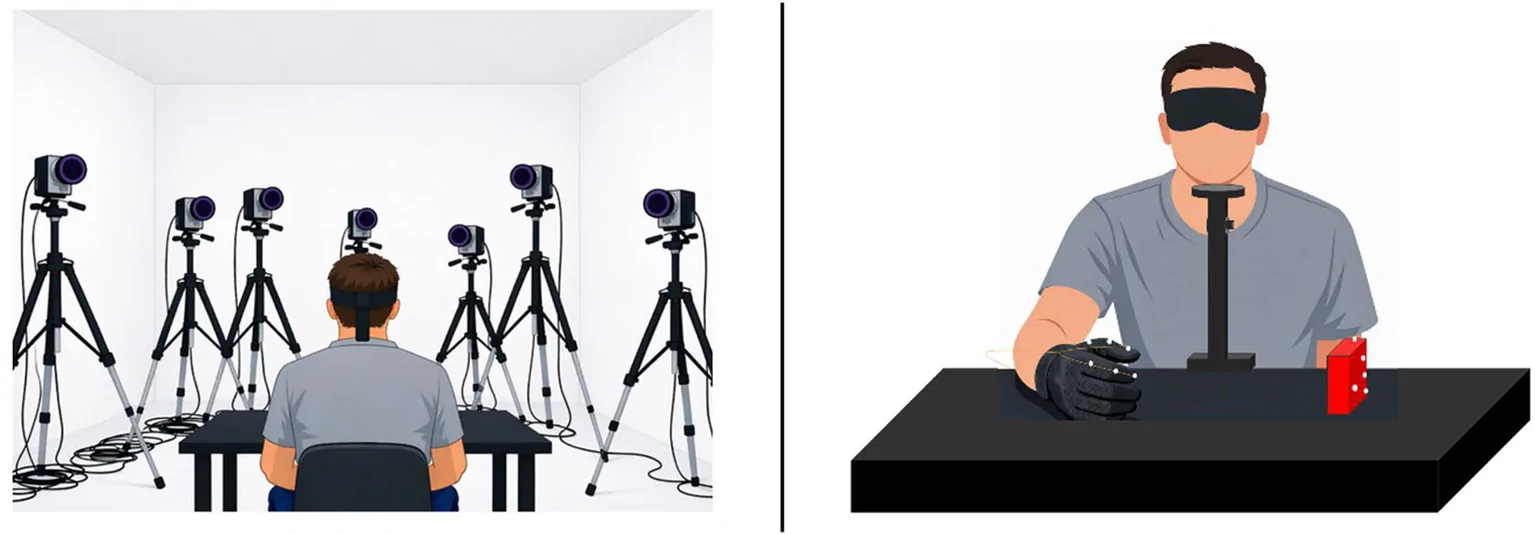
Experimental setup. This figure was created with the assistance of ChatGPT version 5.3.

The experimental setup is illustrated in Figure 1. Apart from the SSG, the setup included a blindfold, a chair, a table, a chinrest, and two sets of rectangular Lego objects. One set of Lego objects, referred to as *initial objects*, was used to establish the initial position of the hand and fingers, and the other set, referred to as *target objects*, consisted of the to-be-grasped objects. Each set included three objects. All Lego objects had a height of 7.0 cm and a depth of 3.2 cm. Based on the width of the objects (3.2, 4.8, or 6.4 cm), they were referred to as small, medium, or large. Two marker configurations were used to differentiate the initial and target objects with the Qualisys software.

### Procedure

Participants received oral and written instructions. Once seated at the table with their head resting on the chinrest, the experimenter covered the participants’ eyes with a blindfold. Before each trial, the experimenter placed the initial and target objects at the appropriate positions on the table (see next subsection) and guided the right hand of the participant to the initial object. The initial object was later removed. After this removal, the experimenter gave the verbal instruction “you may start now” to signal the beginning of a trial. Participants were informed that, depending on the positions and pointing directions of the fingers, in most cases they would need to move their hand in order to generate vibration. The aim of participants was to reach to and grasp the target object. Once the target object was grasped or the hand had passed it, the trial ended, and the motors stopped vibrating.

In addition to the reaching and grasping task, participants provided subjective estimates of the task load at four times during the experiment (after each pretest and posttest), using a pencil-and-paper version of a Spanish translation of the unweighted NASA-TLX (Hart, 2006). Given that we did not consider the subjective estimates sufficiently informative, they are not reported in the Results section. Instead, ANOVAs on the individual NASA-TLX dimensions and on the average scores are reported in Supplementary Supplementary Tables 1–7. The condition means and standard deviations of these variables can be found in Supplementary Supplementary Table 8.

### Experimental design

To describe the design, we first need to explain the possible object positions. In the following, the *x* coordinate refers to the direction parallel to the participant’s frontal plane and the *y* coordinate to the direction parallel to the sagittal plane. The initial and target objects were always 40 cm apart in the *x* direction, with the initial object being placed about 10 cm to the right of the participant and the target object about 30 cm to the left. The movement of the hand thus went from the right to the left (as seen from the perspective of the participant). The positions of the objects along the *y* axis varied, with three possible placements: near, middle, and far (i.e., with a *y* distance of about 10, 15, and 20 cm between the object and the participant). Additionally, as described above, both types of objects could have three different sizes. An experimental trial was thus fully characterized by four parameters (that could each have one of three values): the *y* positions of the initial and target objects and sizes of these same objects.

**Figure 2:**
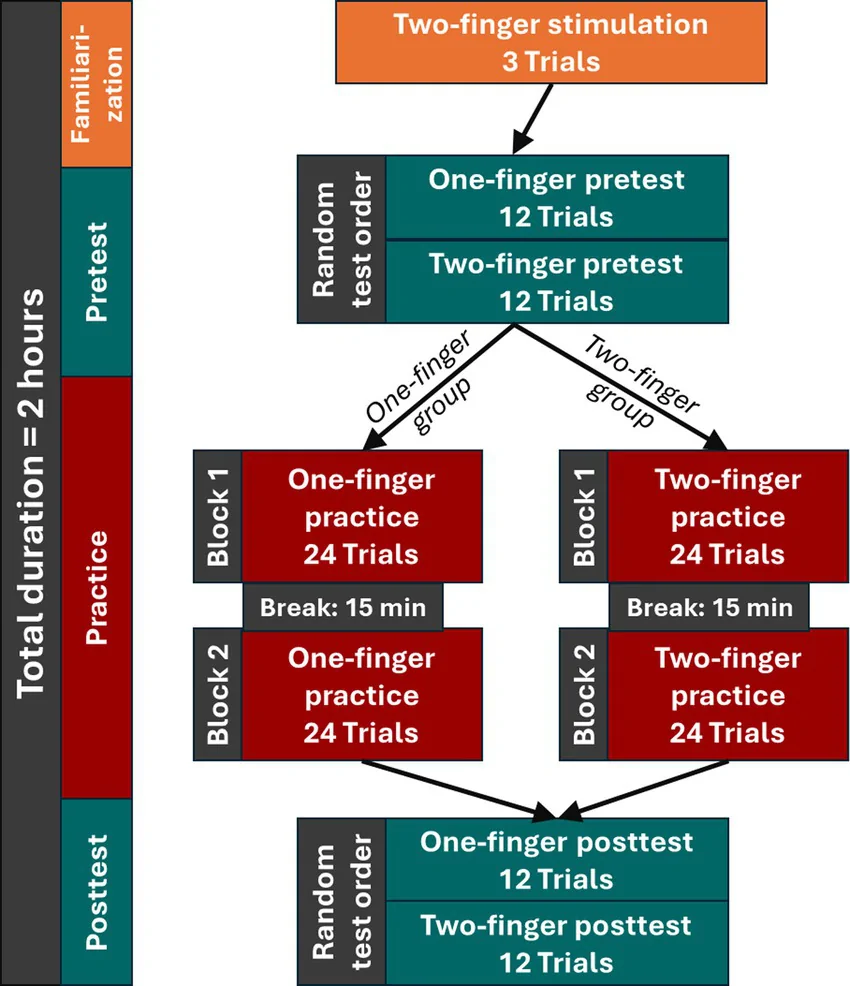
Flow diagram of experimental design.

As can be seen in Figure 2, the experiment was divided into four phases. These phases were the familiarization phase (3 trials), pretest (12 trials with one-finger stimulation and 12 trials with two-finger stimulation), practice (48 trials with either one-finger or two-finger stimulation, depending on the group), and posttest (12 trials with one-finger stimulation and 12 trials with two-finger stimulation). Apart from the practice phase, all phases were the same for the one-finger and two-finger groups. The experiment was completed in approximately 2 h, including a 15-min break in the middle of the practice phase. We next provide a more detailed description of the phases.

The familiarization phase consisted of three trials with the medium-size initial and target objects placed at the middle positions, performed with vibrotactile stimulation on the index finger and thumb. Participants were not blindfolded in the familiarization phase. Only two-finger stimulation was used, because this was sufficient to introduce participants to the task and to the general functioning of the SSG, while allowing the familiarization phase to be as short as possible.

The pretest consisted of two parts of 12 trials each, referred to as the one-finger pretest and two-finger pretest. Stimulation was applied to the index finger in the one-finger pretest and to the index finger and thumb in the two-finger pretest. The medium size was used for the initial object and all three sizes for the target object. The near and far positions were used for both objects. The factorial combination of these sizes and positions led to 12 trials (1 initial size × 3 target sizes × 2 initial positions × 2 target positions). The order of the trials was randomized. The posttest was identical to the pretest. The order of the one-finger and two-finger tests was counterbalanced across participants in the pretest and posttest. Counterbalancing was used to minimize possible order effects related to practice, fatigue, or adaptation to the task.

The practice phase comprised 2 blocks of 24 trials each. Apart from the stimulation on one or two fingers, the practice phase was the same for both groups. The small and large initial objects were used as well as target objects of all three sizes. Both types of objects were used at the near and far positions. This led to 24 combinations (2 initial sizes × 3 target sizes × 2 initial positions × 2 target positions), ordered randomly.

### Data processing and statistical analysis

The reported analysis concerned the time-series of the *x* and *y* locations (i.e., the horizontal locations) of the markers on the tip of the index finger and thumb. For these markers and dimensions, the data from the pretest, practice, and posttest were gap-filled and smoothed. The movement initiation was defined as the moment at which the speed of one of the two analyzed markers exceeded 5 m/s and the end of the trial as the moment at which the markers had both reached an *x* position 2 cm before the object. In most trials, this latter moment occurred just before the contact between the fingertips and the target.

Trials were checked and (if necessary) removed in two ways: automatically and through visual inspection. A MATLAB routine automatically removed trials according to three criteria: first, if the time-series for the trial contained one or more gaps of more than 0.5 s; second, if the movement initiation did not occur within the first 5 s after the start of the data registration; third, if the markers had already moved 10 cm in the *x* direction at the moment that was identified as the movement initiation. In addition to these automatic procedures, the time-series were inspected visually, further removing trials if irregularities such as unexpected peaks in the marker locations occurred. In total, less than 1% of the trials were removed. When missing values occurred during a trial, the vibration intensity of the motors was determined using the data from the last frame that was properly registered. From our own experience with the SSG, we believe that the brief and infrequent periods without immediately updated vibrotactile stimulation did not affect the experimental results.

As was done in Bilal et al. (2025a), three types of dependent variables were considered, associated to performance, exploration, and the effectiveness of exploration. With regard to performance, we computed the proportion of successful grasps and the duration of the trials. A first dependent variable that was considered in relation to exploration was the variability of the index finger in the anterior–posterior direction. Said with more precision, we computed the *SD* of the *y* coordinate of the top marker on the index finger for the first 80% of the trajectory. The last 20% of the trajectory was not considered because it was in many cases related to the grasp component of the action, which is characterized by a wide hand opening that arguably does not form part of an exploratory movement. A second exploration-related variable that was considered was the variability in the hand opening. This variable was computed by taking the *SD* of the distance between the markers on the tips of the index finger and thumb, again during the first 80% of the trial. In sum, we computed two exploration-related variables, one related to exploration only with the index finger and one related to exploration with both the index finger and thumb. For both dependent variables, higher values of the *SD* indicate more pronounced exploration.

**Figure 3:**
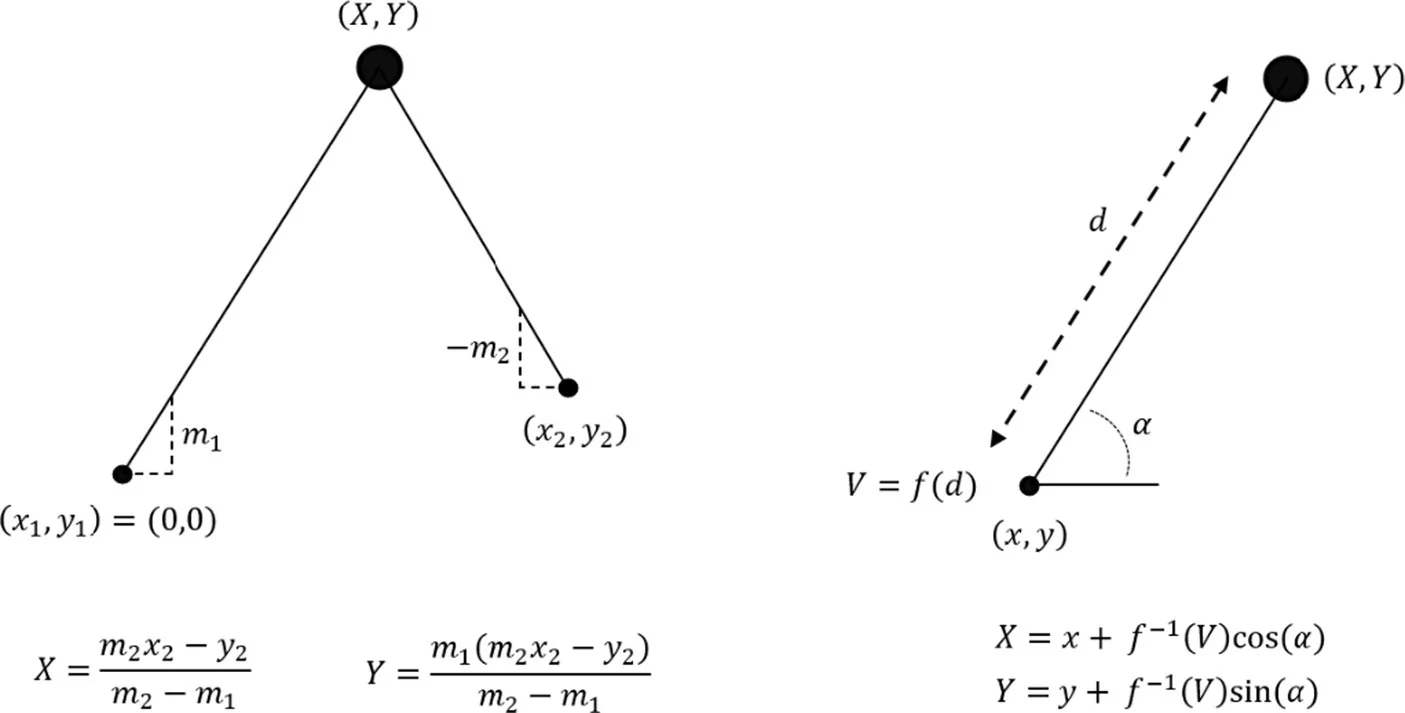
Information about target location that may be detected with SSGs. The properties on the right-hand sides of the equations can potentially be detected with combinations of tactile perception (i.e., the stimulation) and proprioception (i.e., the pointing itself). *X* = x*-*coordinate of target; *Y* = y-coordinate of target; *x*1, *x*2, *x* = x*-*coordinates of points from which the target is detected; *y*1, *y*2, *y* = y-coordinates of points from which the target is detected; *m*1, *m*2 = slopes of lines between points from which the target is detected and target; *α* = angular direction of target; *d* = distance to target; *f* = function that relates target distance to vibration intensity; *V* = vibration intensity. For convenience, the location (*x*1, *y*1) is set at (0, 0).

With regard to the effectiveness of exploration, one could argue that most information is obtained with a finger if the line in the pointing direction of the finger sweeps over the edge of the target. This is so because the on–off (or off–on) switching that occurs during such events (together with the proprioception at that moment) provides information about the edge of the target (Figure 3; further described in Discussion), and the targets are to be grasped at the edges. Based on this reasoning, we referred to a period during which the vibration changed from off to on and then back to off as a vibration bout, and computed the number of bouts of more than 15 ms that occurred per second. This dependent variable was computed for the index finger in the one-finger condition and for both fingers in the two-finger condition. The higher the number of bouts, the more effective the exploration in terms of information detection.

Mixed univariate analyses of variance (ANOVAs) were performed on the dependent variables, for the test-phase data and for the practice-phase data. For the test-phase data, all but one of the dependent variables were analyzed with the factors test phase (pretest, posttest), test condition (one-finger test, two-finger test), and practice condition (one-finger practice, two-finger practice). The exception was the number of vibration bouts for the thumb. Because this variable was computed only in the two-finger condition, we did not use the factor test condition in the corresponding ANOVA. For the practice-phase data, all but one of the dependent variables were analyzed with an ANOVA with the factors practice block (Blocks 1 and 2) and practice condition (one-finger practice, two-finger practice). In this case too, the exception was the number of bouts on the thumb, for which the two practice blocks of the two-finger practice were compared with a single-tailed paired *t-*test (expecting an increase). All significant effects and interactions (*p* < 0.05) of the ANOVAs are reported in the Results section. The full ANOVA results, including the non-significant effects, can be found in Supplementary Supplementary Tables 9–19, and the condition means and standard deviations in Supplementary Supplementary Table 20. For *post-hoc* analyses, Bonferroni corrections were applied to adjust the *p*-values for multiple comparisons.

## Results

In this section we address, in turn, the level of performance, the amount of exploration, and the effectiveness of exploration. Two dependent variables are considered in each subsection.

### Performance

**Figure 4:**
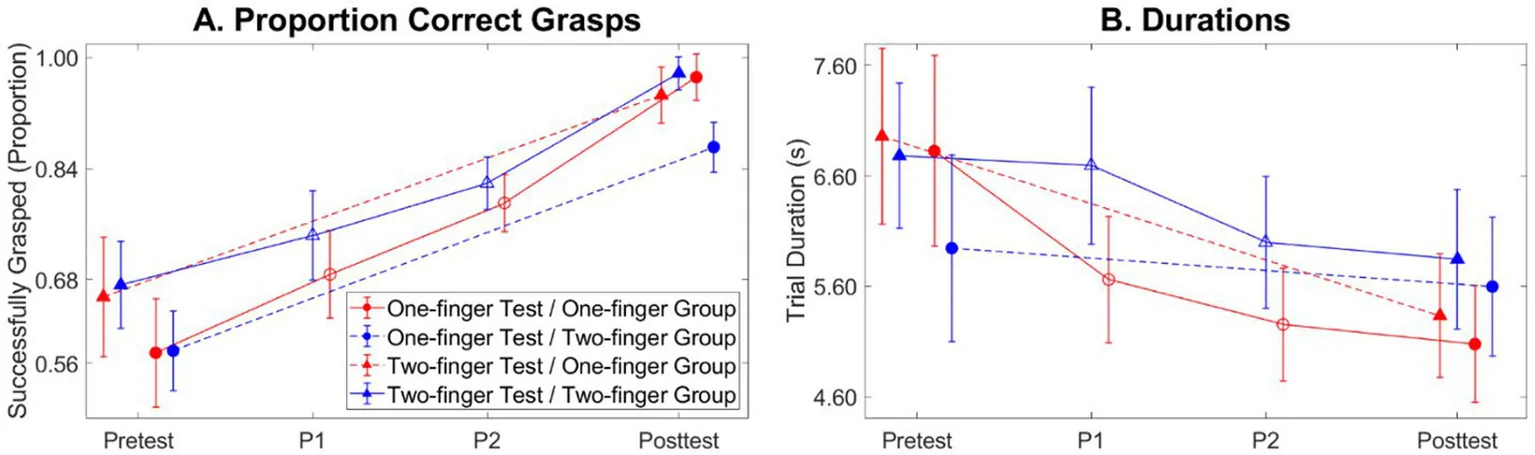
Proportion of successful grasps **(A)**. Trial duration **(B)**. Each panel shows data for the one-finger group (red) and the two-finger group (blue), and for the one-finger tests (circles) and the two-finger tests (triangles). Data from the practice phase is shown if the group performed the corresponding practice. P1 = First practice block; P2 = Second practice block. Error bars represent *SE*s.

The proportions of successful grasps are illustrated in Figure 4A. For the test-phase data, the ANOVA on this variable revealed significant main effects of test phase (*F*[1, 56] = 208.01, *p* < 0.001, $\eta_{G}^{2}$ = 0.055) and test condition (*F*[1, 56] = 24.55, *p* < 0.001, $\eta_{G}^{2}$ = 0.046). Performance improved with practice (in all conditions) and two-finger tests resulted in more successful grasps than one-finger tests. In addition, the Test condition × Practice condition interaction (*F*[1, 56] = 8.06, *p* = 0.006, $\eta_{G}^{2}$ = 0.016) and the Test phase × Test condition × Practice condition interaction (*F*[1, 56] = 4.44, *p* = 0.040, $\eta_{G}^{2}$ = 0.010) were significant. *Post-hoc* comparisons were conducted to further investigate the three-way interaction. The one-finger posttest was performed better by the one-finger group than by the two-finger group (*M*_difference_ = 0.10, *t* = 4.88, *p*_bonf_ < 0.001, *d* = 0.68). In contrast, the performance of the one-finger and two-finger groups did not differ significantly in the two-finger posttest, and neither in the one-finger and two-finger pretests. The ANOVA on the proportion of successful grasps for the practice-phase data revealed a significant effect of practice block (*F*[1, 56] = 22.14, *p* < 0.001, $\eta_{G}^{2}$ = 0.088), demonstrating an increase in performance from Block 1 to Block 2. Overall, these findings indicate that the accuracy of the grasps improved with practice and that transfer of learning occurred between the conditions.

Our second measure of performance, trial duration, is illustrated in Figure 4B. For the test-phase data, the ANOVA revealed a significant main effect of test phase (*F*[1, 56] = 9.41, *p* = 0.003, $\eta_{G}^{2}$ = 0.031), indicating a reduction of the durations from the pretest to the posttest. For the practice-phase data, the main effect of practice block was significant (*F*[1, 56] = 7.72, *p* = 0.007, $\eta_{G}^{2}$ = 0.007), also indicating a reduction in the durations.

### Exploration

**Figure 5:**
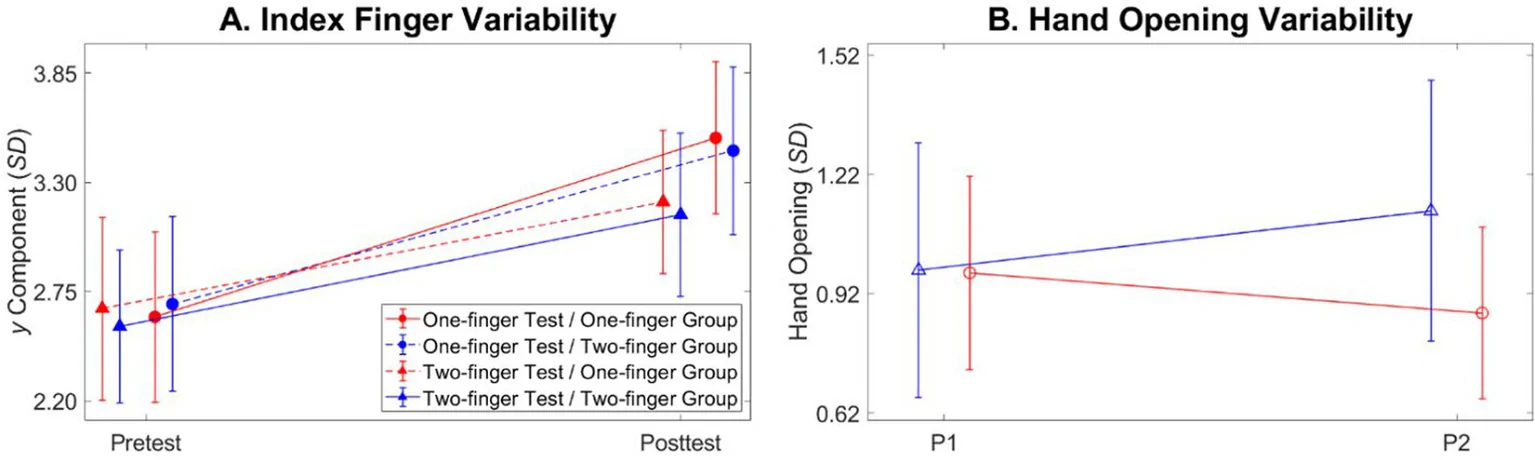
**(A)** *SD* of the *y* component of the top marker on the index finger during the first 80% of the trials. **(B)** *SD* of the hand opening during the first 80% of the trials. Each panel shows data for the one-finger group (red) and the two-finger group (blue), and for the one-finger tests (circles) and the two-finger tests (triangles). Panel A concerns test-phase data and Panel B practice-phase data. P1 = First practice block; P2 = Second practice block. Error bars represent *SE*s.

The most relevant findings with regard to our first measure of exploration, the variability of the tip of the index finger, are illustrated in Figure 5A. For the test-phase data, the ANOVA on this variability revealed significant main effects of test phase (*F*[1, 56] = 51.50, *p* < 0.001, $\eta_{G}^{2}$ = 0.089) and test condition (*F*[1, 56] = 6.12, *p* = 0.016, $\eta_{G}^{2}$ = 0.006) as well as a significant Test phase × Test condition interaction (*F*[1, 56] = 4.38, *p* = 0.041, $\eta_{G}^{2}$ = 0.004). The main effects indicate that the exploration with the index finger was more pronounced after practice and in the one-finger tests. *Post-hoc* comparisons related to the interaction revealed significantly more exploration with the index finger in the one-finger posttests than in the two-finger posttests (*M*_difference_ = 0.30, *t* = 4.18, *p*_bonf_ < 0.001, *d* = 0.27). The ANOVA for the practice-phase data did not reveal significant effects.

The most relevant findings with regard to the variability of the hand opening are illustrated in Figure 5B. For the test-phase data, the ANOVA on this variable revealed significant main effects of test phase (*F*[1, 56] = 10.88, *p* = 0.002, $\eta_{G}^{2}$ = 0.022) and test condition (*F*[1, 56] = 5.99, *p* = 0.017, $\eta_{G}^{2}$ = 0.004). The exploration with the hand opening was more pronounced after practice and in the two-finger tests. The ANOVA on the practice-phase data revealed a significant Practice block × Practice condition interaction (*F*[1, 56] = 5.16, *p* = 0.027, $\eta_{G}^{2}$ = 0.010). As shown in Figure 5B, the exploration with the hand opening decreased during the one-finger practice (red line) and increased during the two-finger practice (blue line).

### Effectiveness of exploration

**Figure 6:**
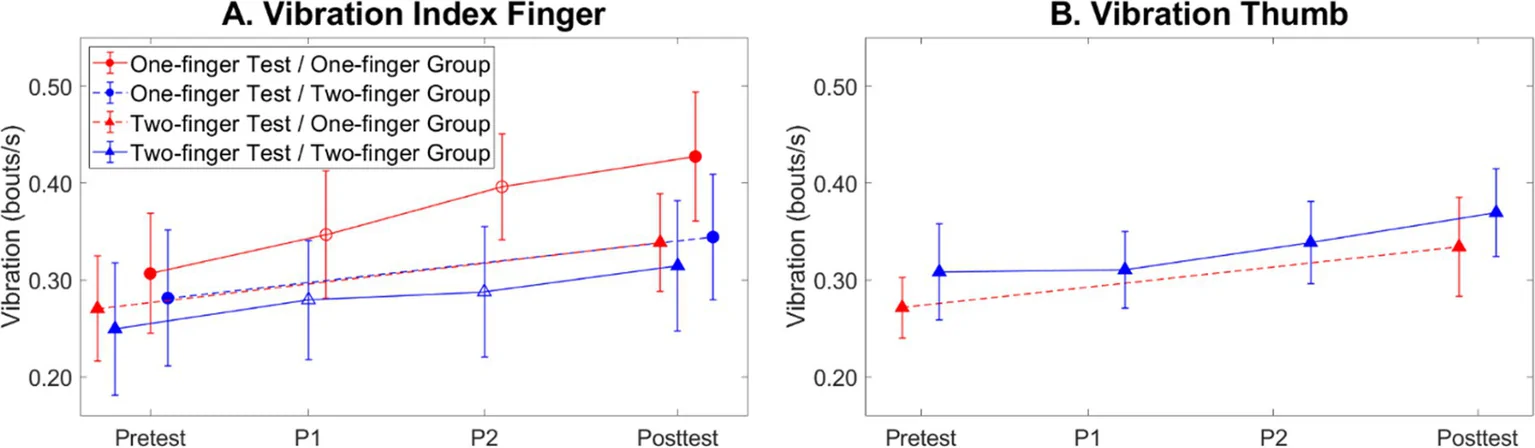
**(A)** Number of vibration bouts of more than 15 ms on the index finger. **(B)** Number of vibration bouts of more than 15 ms on the thumb. Both panels show data for the one-finger group (red) and the two-finger group (blue). The left panel shows data for the one-finger tests (circles) and the two-finger tests (triangles); in contrast, the quantity in the right panel was computed and is shown only for the two-finger tests (triangles). P1 = First practice block; P2 = Second practice block. Error bars represent *SE*s.

Figure 6A shows the number of vibration bouts that were obtained per second with the index finger. Remember that this variable was used as an indication of how effective the exploration was in relation to the information detection. For the test-phase data, the ANOVA revealed significant main effects of test phase (*F*[1, 56] = 18.20, *p* < 0.001, $\eta_{G}^{2}$ = 0.051) and test condition (*F*[1, 56] = 9.04, *p* = 0.004, $\eta_{G}^{2}$ = 0.018). The number of bouts was higher after practice and in the one-finger tests. A *post-hoc* comparison demonstrated that the number of bouts, averaged over the practice conditions, was significantly higher for the one-finger posttest than for the two-finger posttest (*M*_difference_ = 0.059, *t* = 3.30, *p*_bonf_ = 0.010, *d* = 0.34). For the practice-phase data, significant effects were observed for practice block (*F*[1, 56] = 4.78, *p* = 0.033, $\eta_{G}^{2}$ = 0.007) and practice condition (*F*[1, 56] = 4.16, *p* = 0.046, $\eta_{G}^{2}$ = 0.006), also indicating an increase with practice and more bouts in the one-finger condition.

Figure 6B shows the number of vibration bouts for the thumb (in the two-finger condition). For the test phase data, the 2 × 2 ANOVA on this variable revealed a main effect of test phase (*F*[1, 56] = 17.22, *p* < 0.001, $\eta_{G}^{2}$ = 0.061), demonstrating an increase in the number of bouts. For the practice-phase data, the paired *t-*test revealed an increase from Block 1 to Block 2 (*t*[28] = 2.02, *p* = 0.026, *d* = 0.38). The effectiveness of the exploration thus increased with practice.

## Discussion

Previous studies have developed and tested SSGs that indicate with vibration whether or not fingers point to objects. Such SSGs allowed exploration with one finger (Lenay et al., 1997; Lenay et al., 2003) or two fingers (Bilal et al., 2025a; de Paz et al., 2023). The present study examined both modes of functioning. Participants aimed to reach to and grasp targets. The one-finger and two-finger groups practiced with vibration on one or two fingers, respectively. Our most important finding was that the observed improvements with practice transferred between the two configurations: Practice with vibration on one finger facilitated performance with vibration on two fingers, and vice versa. Moreover, as expected on the basis of previous research, individuals showed reasonably high levels of performance in the pretests and further improved with practice. To provide a possible interpretation of the transfer between the modes of functioning, we relate the findings to an ecologically-motivated body of research on the specificity and flexibility of perceptual systems.

In the case of fly ball catching, Bongers and Michaels (2008) argued that perceptual systems detect specific environmental information (optical acceleration) but that they are flexible in *how* the information is detected (using eye or head movements). In the case of dynamic touch, individuals rely on the moments of inertia of the wielded objects with flexibility concerning the anatomical units that are implied (hand, foot, or other; Wagman and Hajnal, 2014; Wagman et al., 2017). To apply this reasoning to the current situation, we assume that lawful patterns exist in the pointing-vibration relations that can be uncovered and exploited with our SSG with vibration provided to either one or two fingers. If so, learning to rely on such informational patterns may entail the development of a functionally-tailored perceptual system that remains flexible in whether the patterns are detected with one or two fingers. The transfer in our experiment is consistent with this interpretation.

The interpretation of the transfer laid out in the previous paragraph requires that informational patterns exist that can be detected with the different configurations of the SSG. Such information must combine proprioceptive information from the pointing action itself and the vibration. But what could that information be? Consider Figure 3. In the figure, the targets are depicted by large filled dots, and their coordinates are given by the uppercase letters *X* and *Y*. Possible positions of the fingertips are given by the small black dots, and their coordinates by lowercase *x*’s and *y*’s. The target on the left is pointed at from two locations and the target on the right from a single location. If adopted with our SSG, such positions would go together with detectable vibration. The equations on the lower left express the target location as a function of the locations ([*x*_1_, *y*_1_], [*x*_2_, *y*_2_]) and pointing directions (*m*_1_, *m*_2_) of the fingers. The equations on the lower right express the target location as a function of the location of the finger (*x*, *y*) and the distance of the target (*d*), which itself is a function of the intensity of vibration (*V*).

The equations on the left of Figure 3 thus demonstrate that, if a target is pointed at (i.e., if vibration occurs), the coordinates of the target are specified by information that is detectable from two finger positions (finger locations and directions to target). Similarly, the equations on the right demonstrate that the coordinates of the target are specified by information that is accessible from a single finger position. The crucial observation at this point is that the informational patterns that are defined in Figure 3 can be detected in different ways. For example, the equations on the left require the target to be pointed at from two points, but it does not matter which two points are used, and neither does it matter whether the points are sampled at the same time (with two fingers) or sequentially (with one finger). The type of information that is portrayed in the figure is hence consistent with the interpretation that individuals learn to exploit specific informational patterns (the geometrical relations given in the equations) while they maintain flexibility in how the patterns are exploited (with one or two fingers).

In addition to the transfer of learning, the experiment demonstrates that performance is more accurate with two fingers than with one. The average proportions of successful grasps for the test phases with vibration on one and two fingers were 0.75 and 0.81, respectively. Different explanations can be given for this finding. First, Bongers and Michaels (2008) argued that the fact that perceptual systems can rely on different mechanisms to detect the same information does not imply that all means of detecting the information are equivalent. In line with this reasoning, even if the same information from the pointing-vibration relations could be exploited with one or two fingers, using two fingers may be more effective. Second, it is possible that, in the two-finger condition, individuals used grasping strategies that require two fingers and therefore cannot be used in the one-finger condition. One such strategy may be the hand-opening strategy, which consists in the opening and closing of the hand while pointing in the direction of the target in order to obtain simultaneous patterns of on–off vibration on the two fingers (see Figure 6 of Bilal et al., 2025a). Both explanations may be partly true. Even so, it should be noted that configuration-specific explanations such as the hand-opening strategy cannot account for the observed transfer. In contrast, the functional explanation based on the flexible detection of configuration-independent relations does allow one to understand the transfer. Also note that one-finger performance was still relatively accurate, especially after practice. As indicated in the Introduction, this may be of practical relevance.

In the reported analysis, positional variability was used as an indication of the amount of exploration with the index finger. This variability increased with practice. Likewise, variability in the hand opening was used as an indication of exploration with the hand-opening strategy. Exploration with this strategy also increased with practice. Our results therefore replicate the overall increase in the amount of exploration that was reported by Bilal et al. (2025a). This is relevant because the increase contrasts with observations by Hacques et al. (2021), who argued that the amount of exploration may decrease with practice if the exploration becomes more effective. We measured the effectiveness of exploration with the number of bouts of vibration that were obtained with a finger. The number of bouts increased with practice, suggesting that the exploration indeed became more effective. With regard to the effectiveness of the exploration, therefore, the current results are in agreement with the results of Bilal et al. as well as with the observations by Hacques et al.

As any other research, this study has limitations. First, we did not include a control group that completed the pretest and posttest without the practice phase. Such a group may have helped to determine how much of the observed improvement was attributable to practice, and whether potential improvements without practice occurred in the same way in the different conditions. Future studies should include such a control group to strengthen the interpretation of the transfer. Second, the familiarization phase used two-finger stimulation only, without blindfolds. This may have given participants some extra proficiency with the two-finger configuration before the formal testing began. We recommend that future studies use a familiarization procedure that is balanced across the one-finger and two-finger modes.

Before we conclude this article, we want to discuss the relation between our findings and another ecologically-motivated body of research on learning. One aspect of learning to perform an action entails changes in which informational variable is detected (Gibson and Gibson, 1955). Whereas beginners often differ in variable use and rely on variables that are not the most useful ones, experts have been shown to graduate to reliance on better information (Fajen and Devaney, 2006; Jacobs et al., 2001). Although we did not track which variables were used at each phase of the learning process, our results are consistent with such a view. The multimodal relations depicted in Figure 3, or similar ones, could constitute information that individuals come to use after practice (while remaining flexible with respect to the fingers that are used for the detection). As an aside, one might argue that explanations of learning that postulate changes in variable use require memory-based processes that are incompatible with the theoretical foundations of the ecological approach. The direct learning theory (Jacobs and Michaels, 2007; cf. Hafkamp and Jacobs, 2026), however, holds that the learning process itself can be direct, hence not requiring such more cognitive processes.

## Conclusion

The present experiment demonstrates that individuals improve in reaching toward and grasping target objects on the basis of an SSG with vibration on one or two fingers and that the improvements transfer between the conditions. Our main conclusion, based on these results, is that the perceptual system that underlies the behavior is functionally specific as well as flexible, meaning that the same pointing-vibration relations can be exploited in different ways. As a final speculation, note that the functional specificity and flexibility of perceptual systems may even go further. That is, individuals may come rely on information that is even more general and modality independent, hence allowing transfer of learning between modalities such as dynamic touch and SSD-based perception (Duffrin and Wagman, 2024) or vision and SSD-based perception (de Paz et al., 2024). If this is the case, then it may be advantageous for SSDs to allow users to rely on information that is in some sense equivalent to information that they have already learned to use with other modalities, allowing the SSD to function as a novel component of a system that is already able to exploit such information, instead of functioning as a novel perceptual system.

## Data Availability

The raw data supporting the conclusions of this article will be made available by the authors, without undue reservation.
