# Supplementary material for: Specificity and flexibility in sensory substitution: learning to grasp with vibration on one or two fingers
Source: Front Psychol. 2026 Sep 3;17:1674806. doi: 10.3389/fpsyg.2026.1674806 (PMC13581827; doi:10.3389/fpsyg.2026.1674806)
Supplement: Supplementary file 1 [file Data_Sheet_1.DOCX]

**Supplementary Table 1**

*ANOVA on NASA-TLX dimension of mental demand*

|  | Sum of squares | *df* | Mean square | *F* | *p* | η²_G_ |
| --- | --- | --- | --- | --- | --- | --- |
| *Within-subjects effects* | | | | | | |
| Test condition | 7.97 | 1 | 7.97 | 1.59 | .213 | 0.002 |
| Test condition × Practice condition | 17.11 | 1 | 17.11 | 3.41 | .070 | 0.005 |
| Test phase | 30.41 | 1 | 30.41 | 3.74 | .058 | 0.008 |
| Test phase × Practice condition | 1.39 | 1 | 1.39 | 0.17 | .680 | 3.9 × 10^-4^ |
| Test condition × Test phase | 2.28 | 1 | 2.28 | 0.51 | .476 | 6.3 × 10^-4^ |
| Test condition × Test phase × Practice condition | 12.11 | 1 | 12.11 | 2.73 | .104 | 0.003 |
| *Between-subjects effect* | | | | | | |
| Practice condition | 70.62 | 1 | 70.62 | 1.51 | .225 | 0.019 |

*Note.* Type III sum of squares was used; *df*_error_ = 56 for all effects.

**Supplementary Table 2**

*ANOVA on NASA-TLX dimension of physical demand*

|  | Sum of squares | *df* | Mean square | *F* | *p* | η²_G_ |
| --- | --- | --- | --- | --- | --- | --- |
| *Within-subjects effects* | | | | | | |
| Test condition | 0.39 | 1 | 0.39 | 0.19 | .661 | 2.3 × 10^-4^ |
| Test condition × Practice condition | 0.57 | 1 | 0.57 | 0.28 | .596 | 3.3 × 10^-4^ |
| Test phase | 23.29 | 1 | 23.29 | 4.17 | .046 | 0.013 |
| Test phase × Practice condition | 10.14 | 1 | 10.14 | 1.81 | .183 | 0.006 |
| Test condition × Test phase | 0.001 | 1 | 0.001 | 5.3 × 10^-4^ | .982 | 6.3 × 10^-7^ |
| Test condition × Test phase × Practice condition | 2.59 | 1 | 2.59 | 1.27 | .263 | 0.001 |
| *Between-subjects effect* | | | | | | |
| Practice condition | 1.32 | 1 | 1.32 | 0.06 | .804 | 7.5 × 10^-4^ |

*Note.* Type III sum of squares was used; *df*_error_ = 56 for all effects.

**Supplementary Table 3**

*ANOVA on NASA-TLX dimension of temporal demand*

|  | Sum of squares | *df* | Mean square | *F* | *p* | η²_G_ |
| --- | --- | --- | --- | --- | --- | --- |
| *Within-subjects effects* | | | | | | |
| Test condition | 2.77 | 1 | 2.77 | 0.70 | .405 | 0.001 |
| Test condition × Practice condition | 10.61 | 1 | 10.61 | 2.68 | .107 | 0.004 |
| Test phase | 64.57 | 1 | 64.57 | 10.57 | .002 | 0.023 |
| Test phase × Practice condition | 1.62 | 1 | 1.62 | 0.26 | .608 | 6.0 × 10^-4^ |
| Test condition × Test phase | 0.92 | 1 | 0.92 | 0.21 | .646 | 3.4 × 10^-4^ |
| Test condition × Test phase × Practice condition | 1.65 | 1 | 1.65 | 0.38 | .538 | 6.1 × 10^-4^ |
| *Between-subjects effect* | | | | | | |
| Practice condition | 12.85 | 1 | 12.85 | 0.37 | .542 | 0.005 |

*Note.* Type III sum of squares was used; *df*_error_ = 56 for all effects.

**Supplementary Table 4**

*ANOVA on NASA-TLX dimension of performance*

|  | Sum of squares | *df* | Mean square | *F* | *p* | η²_G_ |
| --- | --- | --- | --- | --- | --- | --- |
| *Within-subjects effects* | | | | | | |
| Test condition | 4.28 | 1 | 4.28 | 0.80 | .375 | 0.002 |
| Test condition × Practice condition | 6.06 | 1 | 6.06 | 1.13 | .292 | 0.002 |
| Test phase | 42.67 | 1 | 42.67 | 4.79 | .033 | 0.016 |
| Test phase × Practice condition | 25.89 | 1 | 25.89 | 2.90 | .094 | 0.010 |
| Test condition × Test phase | 15.26 | 1 | 15.26 | 3.22 | .078 | 0.006 |
| Test condition × Test phase × Practice condition | 6.06 | 1 | 6.06 | 1.28 | .263 | 0.002 |
| *Between-subjects effect* | | | | | | |
| Practice condition | 0.027 | 1 | 0.027 | 9.9 × 10^-4^ | .975 | 1.0 × 10^-5^ |

*Note.* Type III sum of squares was used; *df*_error_ = 56 for all effects.

**Supplementary Table 5**

*ANOVA on NASA-TLX dimension of effort*

|  | Sum of squares | *df* | Mean square | *F* | *p* | | η²_G_ |
| --- | --- | --- | --- | --- | --- | --- | --- |
| *Within-subjects effects* | | | | | | | |
| Test condition | 0.004 | 1 | 0.004 | 8.0 × 10^-4^ | | .977 | 1.1 × 10^-6^ |
| Test condition × Practice condition | 0.004 | 1 | 0.004 | 8.0 × 10^-4^ | | .977 | 1.1 × 10^-6^ |
| Test phase | 43.97 | 1 | 43.97 | 5.39 | | .024 | 0.011 |
| Test phase × Practice condition | 0.04 | 1 | 0.04 | 0.005 | | .945 | 9.6 × 10^-6^ |
| Test condition × Test phase | 0.11 | 1 | 0.11 | 0.03 | | .853 | 2.7 × 10^-5^ |
| Test condition × Test phase × Practice condition | 3.14 | 1 | 3.14 | 1.01 | | .319 | 7.8 × 10^-4^ |
| *Between-subjects effect* | | | | | | | |
| Practice condition | 61.04 | 1 | 61.04 | 1.10 | .298 | | 0.015 |

*Note.* Type III sum of squares was used; *df*_error_ = 56 for all effects.

**Supplementary Table 6**

*ANOVA on NASA-TLX dimension of frustration*

|  | Sum of squares | *df* | Mean square | *F* | *p* | | η²_G_ |
| --- | --- | --- | --- | --- | --- | --- | --- |
| *Within-subjects effects* | | | | | | | |
| Test condition | 0.35 | 1 | 0.35 | 0.08 | .775 | | 9.9 × 10^-5^ |
| Test condition × Practice condition | 1.10 | 1 | 1.10 | 0.26 | .611 | | 3.1 × 10^-4^ |
| Test phase | 1.25 | 1 | 1.25 | 0.14 | .712 | | 3.5 × 10^-4^ |
| Test phase × Practice condition | 1.72 | 1 | 1.72 | 0.19 | .664 | | 4.9 × 10^-4^ |
| Test condition × Test phase | 0.04 | 1 | 0.04 | 0.01 | .918 | | 1.1 × 10^-5^ |
| Test condition × Test phase × Practice condition | 2.91 | 1 | 2.91 | 0.79 | .375 | | 8.3 × 10^-4^ |
| *Between-subjects effect* | | | | | | | |
| Practice condition | 2.48 | 1 | 2.48 | 0.05 | | .817 | 7.0 × 10^-5^ |

*Note.* Type III sum of squares was used; *df*_error_ = 56 for all effects.

**Supplementary Table 7**

*ANOVA on NASA-TLX average subjective load*

|  | Sum of squares | *df* | Mean square | *F* | *p* | η²_G_ |
| --- | --- | --- | --- | --- | --- | --- |
| *Within-subjects effects* | | | | | | |
| Test condition | 1.23 | 1 | 1.23 | 0.77 | .385 | 8.0 × 10^-4^ |
| Test condition × Practice condition | 0.31 | 1 | 0.31 | 0.19 | .664 | 2.0 × 10^-4^ |
| Test phase | 0.34 | 1 | 0.34 | 0.15 | .703 | 2.3 × 10^-4^ |
| Test phase × Practice condition | 1.49 | 1 | 1.49 | 0.64 | .428 | 9.7 × 10^-4^ |
| Test condition × Test phase | 1.31 | 1 | 1.31 | 1.28 | .263 | 8.6 × 10^-4^ |
| Test condition × Test phase × Practice condition | 0.44 | 1 | 0.44 | 0.43 | .515 | 2.9 × 10^-4^ |
| *Between-subjects effect* | | | | | | |
| Practice condition | 14.30 | 1 | 14.30 | 0.64 | .427 | 0.009 |

*Note.* Type III sum of squares was used; *df*_error_ = 56 for all effects.
