# Supplementary material for: Specificity and flexibility in sensory substitution: learning to grasp with vibration on one or two fingers
Source: Front Psychol. 2026 Sep 3;17:1674806. doi: 10.3389/fpsyg.2026.1674806 (PMC13581827; doi:10.3389/fpsyg.2026.1674806)
Supplement: Supplementary file 2 [file Data_Sheet_2.DOCX]

**Supplementary Table 8**

*Means (M) and Standard Deviations (SD) for individual dimensions and average score of NASA-TLX*

| **Variable** | **Practice group** | **Pretest** | | **Posttest** | |
| --- | --- | --- | --- | --- | --- |
|  |  | One-finger test  *M (SD)* | Two-finger test  *M (SD)* | One-finger test  *M (SD)* | Two-finger test  *M (SD)* |
| *Mental demand* | |  |  |  |  |
|  | One-finger | 5.67 (3.64) | 6.13 (4.04) | 6.81 (3.42) | 6.72 (3.36) |
|  | Two-finger | 7.31 (4.45) | 6.36 (3.79) | 7.85 (4.04) | 7.59 (4.25) |
| *Physical demand* | |  |  |  |  |
|  | One-finger | 4.57 (2.31) | 4.56 (2.48) | 4.96 (2.37) | 4.57 (2.75) |
|  | Two-finger | 4.37 (2.68) | 4.19 (2.32) | 5.22 (3.54) | 5.45 (3.43) |
| *Temporal demand* | |  |  |  |  |
|  | One-finger | 6.48 (3.93) | 5.87 (3.22) | 5.64 (3.12) | 4.95 (2.95) |
|  | Two-finger | 6.86 (4.28) | 6.78 (3.68) | 5.35 (2.99) | 5.85 (3.45) |
| *Performance* | |  |  |  |  |
|  | One-finger | 7.62 (4.74) | 6.19 (3.55) | 5.25 (3.12) | 5.50 (3.52) |
|  | Two-finger | 6.32 (3.46) | 6.19 (3.16) | 5.95 (2.54) | 6.19 (2.55) |
| *Effort* | |  |  |  |  |
|  | One-finger | 6.51 (4.35) | 6.70 (3.91) | 7.55 (4.32) | 7.36 (3.72) |
|  | Two-finger | 7.76 (4.36) | 7.46 (4.17) | 8.37 (4.38) | 8.63 (4.64) |
| *Frustration* | |  |  |  |  |
|  | One-finger | 5.94 (4.48) | 5.63 (3.88) | 5.67 (3.82) | 5.86 (3.55) |
|  | Two-finger | 5.62 (4.24) | 6.03 (3.52) | 6.13 (3.95) | 6.16 (4.17) |
| *Average score* | |  |  |  |  |
|  | One-finger | 6.13 (2.82) | 5.85 (2.63) | 5.98 (2.46) | 5.82 (2.43) |
|  | Two-finger | 6.48 (2.69) | 6.17 (2.43) | 6.48 (2.59) | 6.64 (2.88) |
