# Supplementary material for: Specificity and flexibility in sensory substitution: learning to grasp with vibration on one or two fingers
Source: Front Psychol. 2026 Sep 3;17:1674806. doi: 10.3389/fpsyg.2026.1674806 (PMC13581827; doi:10.3389/fpsyg.2026.1674806)
Supplement: Supplementary file 3 [file Data_Sheet_3.DOCX]

**Supplementary Table 9**

*ANOVA on proportion of successful grasps for test-phase data*

|  | Sum of squares | *df* | Mean square | *F* | *p* | η²_G_ |
| --- | --- | --- | --- | --- | --- | --- |
| *Within-subjects effects* | | | | | | |
| Test condition | 0.24 | 1 | 0.24 | 24.55 | < .001 | 0.046 |
| Test condition × Practice condition | 0.08 | 1 | 0.08 | 8.06 | .006 | 0.016 |
| Test phase | 5.98 | 1 | 5.98 | 208.01 | < .001 | 0.055 |
| Test phase × Practice condition | 0.03 | 1 | 0.03 | 1.00 | .322 | 0.006 |
| Test condition × Test phase | 0.03 | 1 | 0.03 | 2.88 | .095 | 0.007 |
| Test condition × Test phase × Practice condition | 0.05 | 1 | 0.05 | 4.44 | .040 | 0.010 |
| *Between-subjects effect* | | | | | | |
| Practice condition | 0.01 | 1 | 0.01 | 0.23 | .632 | 0.002 |

*Note.* Type III sum of squares was used; *df*_error_ = 56 for all effects.

**Supplementary Table 10**

*ANOVA on proportion of successful grasps for practice-phase data*

|  | Sum of squares | *df* | Mean square | *F* | *p* | η²_G_ |
| --- | --- | --- | --- | --- | --- | --- |
| *Within-subjects effects* | | | | | | |
| Practice phase | 0.23 | 1 | 0.23 | 22.14 | < .001 | 0.088 |
| Test condition × Practice condition | 0.01 | 1 | 0.01 | 0.533 | .468 | 0.002 |
| *Between-subjects effect* | | | | | | |
| Practice condition | 0.05 | 1 | 0.05 | 1.614 | .209 | 0.021 |

*Note.* Type III sum of squares was used; *df*_error_ = 56 for all effects.

**Supplementary Table 11**

*ANOVA on trial duration for test-phase data*

|  | Sum of squares | *df* | Mean square | *F* | *p* | η²_G_ |
| --- | --- | --- | --- | --- | --- | --- |
| *Within-subjects effects* | | | | | | |
| Test condition | 7.91 | 1 | 7.91 | 3.25 | .077 | 0.003 |
| Test condition × Practice condition | 1.74 | 1 | 1.74 | 0.72 | .400 | 7.2 × 10^-4^ |
| Test phase | 78.74 | 1 | 78.74 | 9.41 | .003 | 0.031 |
| Test phase × Practice condition | 15.84 | 1 | 15.84 | 1.89 | .175 | 0.006 |
| Test condition × Test phase | 0.79 | 1 | 0.79 | 0.45 | .505 | 3.2 × 10^-4^ |
| Test condition × Test phase × Practice condition | 1.84 | 1 | 1.89 | 1.06 | .308 | 7.6 × 10^-4^ |
| *Between-subjects effect* | | | | | | |
| Practice condition | 0.002 | 1 | 0.002 | 6.9 × 10^-5^ | .993 | 8.8 × 10^-7^ |

*Note.* Type III sum of squares was used; *df*_error_ = 56 for all effects.

**Supplementary Table 12**

*ANOVA on trial duration for practice-phase data*

|  | Sum of squares | *df* | Mean square | *F* | *p* | η²_G_ |
| --- | --- | --- | --- | --- | --- | --- |
| *Within-subjects effects* | | | | | | |
| Practice phase | 8.82 | 1 | 8.82 | 7.72 | .007 | 0.007 |
| Test condition × Practice condition | 0.60 | 1 | 0.60 | 0.53 | .468 | 5.1 × 10^-4^ |
| *Between-subjects effect* | | | | | | |
| Practice condition | 22.91 | 1 | 22.91 | 1.33 | .292 | 0.019 |

*Note.* Type III sum of squares was used; *df*_error_ = 56 for all effects.

**Supplementary Table 13**

*ANOVA on index finger variability for test-phase data*

|  | Sum of squares | *df* | Mean square | *F* | *p* | η²_G_ |
| --- | --- | --- | --- | --- | --- | --- |
| *Within-subjects effects* | | | | | | |
| Test condition | 1.64 | 1 | 1.64 | 6.12 | .016 | 0.006 |
| Test condition × Practice condition | 0.04 | 1 | 0.04 | 0.17 | .670 | 1.6 × 10^-4^ |
| Test phase | 26.95 | 1 | 26.95 | 51.50 | < .001 | 0.089 |
| Test phase × Practice condition | 0.08 | 1 | 0.08 | 0.15 | .699 | 2.9 × 10^-4^ |
| Test condition × Test phase | 1.03 | 1 | 1.03 | 4.38 | .041 | 0.004 |
| Test condition × Test phase × Practice condition | 0.14 | 1 | 0.14 | 0.59 | .445 | 5.0 × 10^-4^ |
| *Between-subjects effect* | | | | | | |
| Practice condition | 1.62 | 1 | 1.62 | 0.42 | .520 | 0.006 |

*Note.* Type III sum of squares was used; *df*_error_ = 56 for all effects.

**Supplementary Table 14**

*ANOVA on index finger variability for practice-phase data*

|  | Sum of squares | *df* | Mean square | *F* | *p* | η²_G_ |
| --- | --- | --- | --- | --- | --- | --- |
| *Within-subjects effects* | | | | | | |
| Practice phase | 0.46 | 1 | 0.46 | 3.27 | .070 | 0.003 |
| Test condition × Practice condition | 4.6 × 10^-5^ | 1 | 4.6 × 10^-5^ | 3.0 × 10^-3^ | .980 | 3.2 × 10^-7^ |
| *Between-subjects effect* | | | | | | |
| Practice condition | 1.23 | 1 | 1.23 | 0.49 | .483 | 0.008 |

*Note.* Type III sum of squares was used; *df*_error_ = 56 for all effects.

**Supplementary Table 15**

*ANOVA on hand-opening variability for test-phase data*

|  | Sum of squares | *df* | Mean square | *F* | *p* | η²_G_ |
| --- | --- | --- | --- | --- | --- | --- |
| *Within-subjects effects* | | | | | | |
| Test condition | 0.55 | 1 | 0.55 | 5.99 | .017 | 0.004 |
| Test condition × Practice condition | 0.01 | 1 | 0.01 | 0.13 | .722 | 9.6 × 10^-5^ |
| Test phase | 2.77 | 1 | 2.77 | 10.88 | .002 | 0.022 |
| Test phase × Practice condition | 0.67 | 1 | 0.67 | 2.66 | .109 | 0.005 |
| Test condition × Test phase | 0.003 | 1 | 0.003 | 0.03 | .873 | 2.3 × 10^-5^ |
| Test condition × Test phase × Practice condition | 0.002 | 1 | 0.002 | 0.02 | .901 | 1.4 × 10^-5^ |
| *Between-subjects effect* | | | | | | |
| Practice condition | 0.09 | 1 | 0.09 | 0.05 | .820 | 7.6 × 10^-4^ |

*Note.* Type III sum of squares was used; *df*_error_ = 56 for all effects.

**Supplementary Table 16**

*ANOVA on hand-opening variability for practice-phase data*

|  | Sum of squares | *df* | Mean square | *F* | *p* | η²_G_ |
| --- | --- | --- | --- | --- | --- | --- |
| *Within-subjects effects* | | | | | | |
| Practice phase | 0.07 | 1 | 0.07 | 0.54 | .467 | 0.001 |
| Test condition × Practice condition | 0.65 | 1 | 0.65 | 5.16 | .027 | 0.010 |
| *Between-subjects effect* | | | | | | |
| Practice condition | 0.50 | 1 | 0.50 | 0.51 | .477 | 0.008 |

*Note.* Type III sum of squares was used; *df*_error_ = 56 for all effects.

**Supplementary Table 17**

*ANOVA on vibration bouts on index finger for test-phase data*

|  | Sum of squares | *df* | Mean square | *F* | *p* | η²_G_ |
| --- | --- | --- | --- | --- | --- | --- |
| *Within-subject effects* | | | | | | |
| Test condition | 0.13 | 1 | 0.13 | 9.04 | .004 | 0.018 |
| Test condition × Practice condition | 0.02 | 1 | 0.02 | 1.05 | .309 | 0.002 |
| Test phase | 0.36 | 1 | 0.36 | 18.20 | < .001 | 0.051 |
| Test phase × Practice condition | 0.01 | 1 | 0.01 | 0.67 | .417 | 0.002 |
| Test condition × Test phase | 0.01 | 1 | 0.01 | 1.08 | .304 | 0.001 |
| Test condition × Test phase × Practice condition | 0.01 | 1 | 0.01 | 1.26 | .266 | 0.002 |
| *Between-subjects effect* | | | | | | |
| Practice condition | 0.09 | 1 | 0.09 | 1.086 | .302 | 0.012 |

*Note.* Type III sum of squares was used; *df*_error_ = 56 for all effects.

**Supplementary Table 18**

*ANOVA on vibration bouts on index finger for practice-phase data*

|  | Sum of squares | *df* | Mean square | *F* | *p* | η²_G_ |
| --- | --- | --- | --- | --- | --- | --- |
| *Within-subjects effects* | | | | | | |
| Practice phase | 0.02 | 1 | 0.02 | 4.78 | .033 | 0.007 |
| Test condition × Practice condition | 0.01 | 1 | 0.01 | 2.43 | .124 | 0.004 |
| *Between-subjects effect* | | | | | | |
| Practice condition | 0.22 | 1 | 0.22 | 4.16 | .046 | 0.006 |

*Note.* Type III sum of squares was used; *df*_error_ = 56 for all effects.

**Supplementary Table 19**

*ANOVA on vibration bouts on thumb for test-phase data*

|  | Sum of squares | *df* | Mean square | *F* | *p* | η²_G_ |
| --- | --- | --- | --- | --- | --- | --- |
| *Within-subjects effects* | | | | | | |
| Test phase | 0.11 | 1 | 0.11 | 17.22 | < .001 | 0.061 |
| Test phase × Practice condition | 1.2 × 10^-5^ | 1 | 1.2 × 10^-5^ | 0.002 | .966 | 7.1 × 10^-6^ |
| *Between-subjects effect* | | | | | | |
| Practice condition | 0.04 | 1 | 0.04 | 1.57 | .216 | 0.022 |

*Note.* Type III sum of squares was used; *df*_error_ = 56 for all effects.
