# Supplementary material for: Specificity and flexibility in sensory substitution: learning to grasp with vibration on one or two fingers
Source: Front Psychol. 2026 Sep 3;17:1674806. doi: 10.3389/fpsyg.2026.1674806 (PMC13581827; doi:10.3389/fpsyg.2026.1674806)
Supplement: Supplementary file 4 [file Data_Sheet_4.DOCX]

**Supplementary Table 20**

*Means (M) and Standard Deviations (SD) for all dependent variables*

| **Variable** | **Practice group** | **Pretest** | | **Practice** | | **Posttest** | |
| --- | --- | --- | --- | --- | --- | --- | --- |
|  |  | One-finger test  *M (SD)* | Two-finger test  *M (SD)* | Block 1  *M (SD)* | Block 2  *M (SD)* | One-finger test  *M (SD)* | Two-finger test  *M (SD)* |
| *Proportion successful*  *grasps* | |  |  |  |  |  |  |
|  | One-finger | 0.58 (0.22) | 0.66 (0.24) | 0.69 (0.17) | 0.79 (0.12) | 0.97 (0.05) | 0.95 (0.06) |
|  | Two-finger | 0.58 (0.16) | 0.67 (0.17) | 0.74 (0.18) | 0.82 (0.11) | 0.87 (0.09) | 0.98 (0.04) |
| *Trial duration* | |  |  |  |  |  |  |
|  | One-finger | 6.82 (3.87) | 6.96 (3.57) | 5.66 (2.57) | 5.26 (2.28) | 5.08 (2.37) | 5.33 (2.52) |
|  | Two-finger | 5.94 (3.04) | 6.79 (3.93) | 6.69 (4.25) | 5.99 (3.58) | 5.59 (2.83) | 5.84 (3.78) |
| *Variability index*  *finger* | |  |  |  |  |  |  |
|  | One-finger | 2.62 (1.18) | 2.66 (1.27) | 3.16 (1.30) | 3.28 (1.51) | 3.53 (1.05) | 3.20 (0.98) |
|  | Two-finger | 2.57 (1.14) | 2.46 (1.01) | 2.95 (1.05) | 3.08 (1.04) | 3.29 (1.15) | 3.02 (1.08) |
| *Variability hand*  *opening* | |  |  |  |  |  |  |
|  | One-finger | 0.88 (0.62) | 0.99 (0.72) | 0.97 (0.67) | 0.87 (0.59) | 0.99 (0.77) | 1.10 (0.82) |
|  | Two-finger | 0.75 (0.71) | 0.84 (0.74) | 0.95 (0.79) | 1.51 (0.86) | 1.08 (0.77) | 1.15 (0.77) |
| *Vibration bouts on*  *index finger* | |  |  |  |  |  |  |
|  | One-finger | 0.30 (0.17) | 0.27 (0.15) | 0.35 (0.18) | 0.39 (0.15) | 0.43 (0.18) | 0.34 (0.14) |
|  | Two-finger | 0.28 (0.19) | 0.25 (0.19) | 0.28 (0.17) | 0.29 (0.18) | 0.35 (0.18) | 0.32 (0.19) |
| *Vibration bouts on*  *thumb* | |  |  |  |  |  |  |
|  | One-finger |  | 0.27 (0.09) |  |  |  | 0.33 (0.14) |
|  | Two-finger |  | 0.31 (0.14) | 0.31 (0.11) | 0.34 (0.12) |  | 0.37 (0.12) |
